# Psychosocial mediators for the impact of personal genomic risk information on melanoma prevention and early detection behaviors

**DOI:** 10.1093/abm/kaag046

**Published:** 2026-08-26

**Authors:** Sabrina E Wang, David Espinoza, Serigne N Lo, Amelia K Smit, Anne E Cust

**Affiliations:** The Daffodil Centre, The University of Sydney, and Cancer Council NSW, Sydney 2011, Australia; The Daffodil Centre, The University of Sydney, and Cancer Council NSW, Sydney 2011, Australia; NHMRC Clinical Trials Centre, The University of Sydney, Sydney 2050, Australia; Melanoma Institute Australia, The University of Sydney, Sydney 2065, Australia; The Daffodil Centre, The University of Sydney, and Cancer Council NSW, Sydney 2011, Australia; The Daffodil Centre, The University of Sydney, and Cancer Council NSW, Sydney 2011, Australia; Melanoma Institute Australia, The University of Sydney, Sydney 2065, Australia

**Keywords:** melanoma, genomic risk, psychosocial factors, behavior change, mediation analysis

## Abstract

**Importance:**

In the Melanoma Genomics Managing Your Risk Study, receiving personal genomic risk testing led to improvements in some melanoma prevention and early detection behaviors.

**Objective:**

We aimed to examine the hypothesized psychosocial mediators of the effects observed in the trial.

**Design:**

Australians of European ancestry without melanoma and aged 18-69 years were recruited via the national Medicare database and randomized to receive personal genomic risk information or usual care (*N* = 1025). Questionnaires were administered at baseline, 1-month post-intervention, and 12-months post-baseline to assess self-reported prevention and early detection behaviors and psychosocial measures. We first evaluated the intervention’s effect on psychosocial measures and the associations between psychosocial measures and behavioral outcomes. We then estimated the natural indirect effects (NIEs) and their 95% confidence intervals (CIs) to quantify the effects mediated by potential mediators identified.

**Results:**

Among participants with high traditional melanoma risk, the intervention’s effect on increased sun protection at 1 month was partially mediated by changes in the perceived importance (NIE mean difference 0.02; 95% CI: 0.00-0.04) and perceived effectiveness (0.01; 0.00-0.03]) of sun protection strategies. Among women, the intervention’s effect on increased whole-body skin examinations at 1 month was partially mediated by perceived capability to engage in skin examinations (NIE odds ratio 1.08; 95% CI: 1.00-1.29) and perceived control over detecting a future melanoma (1.13; 1.03-1.32).

**Conclusions and Relevance:**

The effectiveness of precision prevention and early detection interventions may be enhanced by targeting key psychosocial mediators through tailored communication of personal melanoma risk.

## Introduction

Reducing exposure to ultraviolet radiation is an important modifiable strategy for melanoma prevention, and early detection of skin cancer is associated with improved outcomes and reduced mortality.^1–3^ Given limited clinical trial evidence that population-based screening reduces melanoma mortality, current guidelines emphasize risk stratification, targeted surveillance, and behavioral counseling for individuals at elevated risk.^1^^,^^4^ Personal genomic risk information may support these approaches by improving risk stratification while also providing a potential avenue for motivating prevention and early detection behaviors.

The Melanoma Genomics Managing Your Risk Study is a randomized controlled trial designed to evaluate the impact of providing personal genomic risk information on melanoma prevention and early detection behaviors in a general Australian population.^5^ The primary trial reported that receiving personal genomic risk information led to improvements in several behavioral outcomes, most evidently among participants with high traditional melanoma risk scores and among women.^5^ As a prespecified secondary analysis of the trial, we investigated theoretically derived psychosocial mediators of the intervention’s effects on melanoma prevention and early detection behaviors.

Understanding the motivations for behavior change is important for advancing precision health communication and optimizing future prevention and early detection interventions. Health behavior theories offer a useful framework for understanding the potential psychosocial pathways through which personal genomic risk information influences health behavior. Specifically, the Protection Motivation Theory^6^ and the Health Belief Model^7^ suggest engagement in preventive and early detection behaviors may be influenced by perceived disease severity and susceptibility, beliefs about the effectiveness and importance of recommended actions, self-efficacy, and perceived barriers. The intervention delivered in the primary trial was guided by these theoretical models and targeted key components that are hypothesized to motivate behavior change within the context of communicating personal genomic risk information. Qualitative research has shown that communicating personal melanoma risk information may increase individuals’ sense of preparedness and ability to manage their cancer risk.^8^ However, evidence on formally quantified psychosocial mechanisms linking personal genomic risk communication to behavior change remains limited. We hypothesized that receiving personal melanoma risk information would influence some of the psychosocial determinants measured, which in turn would mediate the subsequent changes in melanoma prevention and early detection behaviors.

## Methods

### Study design and population

This analysis included participants of the Melanoma Genomics Managing Your Risk Study.^5^ Briefly, eligibility for the trial included age 18-69 years at the time of recruitment, no personal history of melanoma, and full or part European ancestry. Recruitment occurred between October 2017 and February 2019, with 1025 participants completing baseline measures and randomized to receive the intervention (*n* = 513; personalized melanoma risk booklet based on a polygenic risk score, genetic counseling via telephone, educational booklet) or control (*n* = 512; educational booklet). Randomization was stratified by traditional risk phenotype (low, high), sex, state of residence, and age group (18-44, 45-69 years). Hypothesized psychosocial mediators and behavioral outcomes were assessed via questionnaires at baseline, 1 month after intervention (T1), and 12 months after baseline (T2). A total of 994 (97%) participants completed the questionnaire at follow-up^1^ and 973 (95%) participants completed the questionnaire at follow-up.^2^ The detailed study protocol, statistical plan, and main results of the trial are published.^5^^,^^9^^,^^10^ The methods and results of this present analysis are reported following the Strengthening the Reporting of Observational Studies in Epidemiology Statement for cohort studies.^11^

### Behavioral outcomes

**Figure 1:**
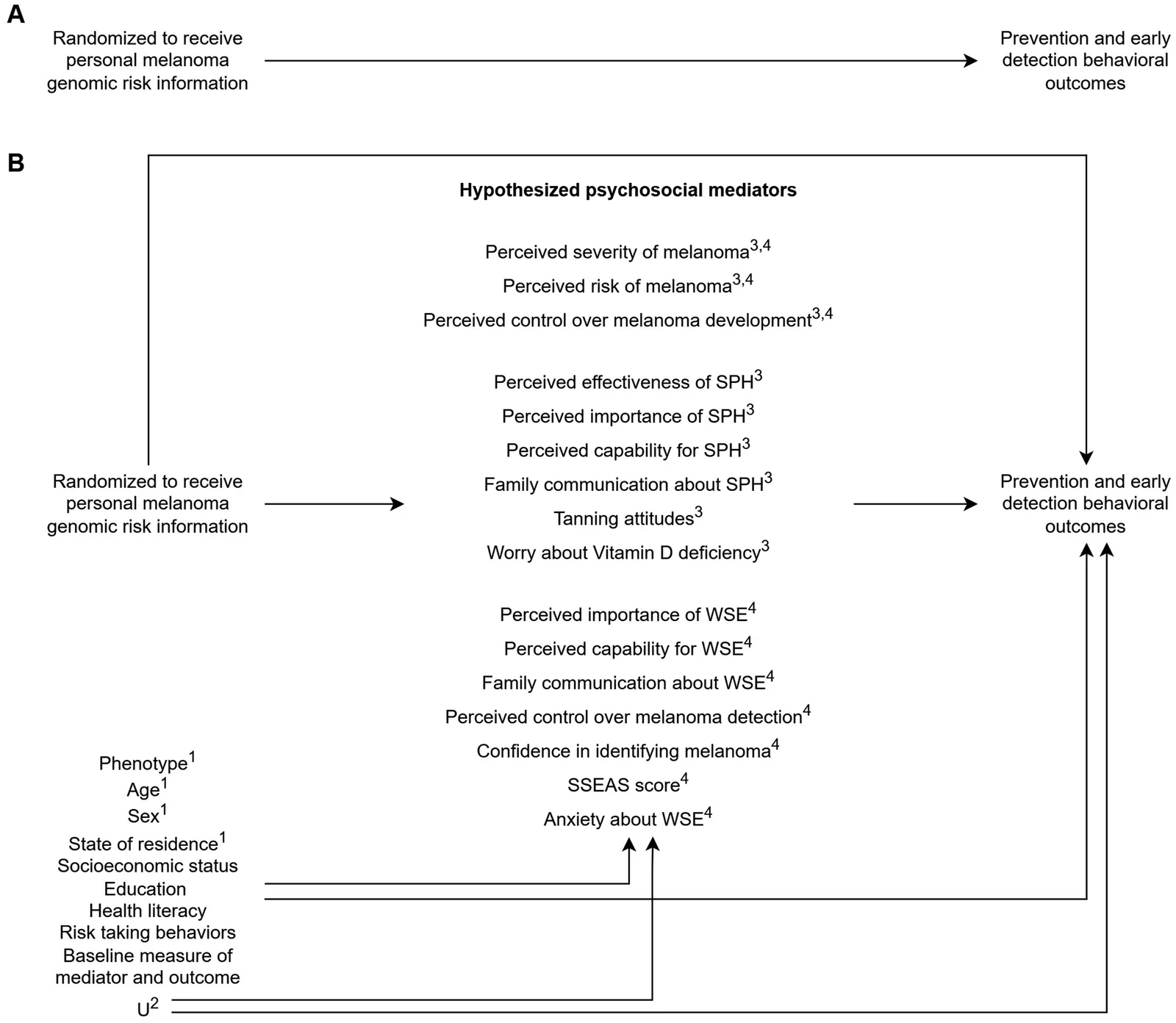
(A) Causal graph illustrating the intervention’s effect on behavioral outcomes in the Melanoma Genomics Managing Your Risk Study trial; (B) causal graph illustrating the mediation analysis aimed to examine hypothesized psychosocial mediators of the effects observed in the trial. ^1^Stratification factors for randomization. ^2^U denotes unassessed confounding factors. ^3^Hypothesized mediators for the intervention’s effect on sun protection outcomes (ie, sun protection habits, intentional tanning, and sunburn). ^4^Hypothesized mediators for the intervention’s effect on early detection behavior (ie, whole-body skin examination). Abbreviations: SPH = sun protection habits; SSEAS = Skin Self-Examination Attitude Scale; WSE = whole-body skin examination.

Outcomes of interest were those demonstrating statistically significant between-group differences (*P* < .05) in the main trial (Figure 1A). These included: (1) improved sun protection habits at T1 among participants in the high traditional risk group; (2) reduced intentional tanning frequency at T1 among women; (3) increased whole-body skin examination at T1 among women; and (4) reduced sunburn at T2 for all participants.

Sun protection habits index was a validated composite score calculated as the mean of 6 sun protection habits adopted in the past month (wearing sunscreen, wearing a shirt with sleeves, wearing a hat, staying in the shade, wearing sunglasses, limiting midday sun exposure), each assessed on a 4-point Likert scale (1 = never or rarely, 2 = sometimes, 3 = often, 4 = always).^12^ Intentional tanning frequency in the past month was assessed on a 5-point Likert scale (1 = never, 2 = rarely, 3 = sometimes; 4 = often, 5 = always). Whole-body skin examination, self-performed or conducted by a health professional, was dichotomized as “yes” (examination on all or nearly all of the skin) or “no” (all other responses). Sunburn frequency in the past month was collected (0, 1, 2, or 3 or more times) and dichotomized as “any” or “none.”

### Hypothesized psychosocial mediators

The development of proposed mediator measures was guided by the constructs of the Protection Motivation Theory^6^ and the Health Belief Model.^7^ We hypothesized that receiving personal melanoma risk information could influence an individual’s perceptions of disease severity, personal susceptibility, as well as their beliefs about the efficacy of, and the ability or barriers to undertake preventive activities, thereby impacting their engagement in health behaviors. The assumed causal structure involving the mediators is illustrated in Figure 1B. For hypothesized mediators referring to sun protection habits, we used the total index score. In an exploratory analysis, we examined individual habits separately (eg, perceived effectiveness of wearing sunscreen as the hypothesized mediator). For hypothesized mediators referring to whole-body skin examinations, we used the mean Likert scale score of those performed by health professionals and those that were self-performed. In an exploratory analysis, we examined self-performed and health professional-performed skin examinations separately (eg, perceived capability to engage in skin self-examination as the hypothesized mediator). More details for each mediator measure are published elsewhere.^9^ We used psychosocial measures collected 1 month post-intervention (at T1).

### Statistical analysis

We first replicated the intervention-outcome effects of interest (Figure 1A) using linear regression to estimate mean differences and 95% confidence interval (CI) for continuous outcomes (sun protection habits index and tanning frequency) and logistic regression to estimate odds ratios and 95% CI for binary outcomes (whole-body skin examination and sunburn). In the main trial, relative risks were reported for binary outcomes. In contrast, odds ratios were used in the present analysis to address potential convergence issues arising from the multiple adjustment factors planned. Estimates obtained from the main trial and from this analysis are presented side-by-side in Table S1.

For each hypothesized mediator identified *a priori*, we evaluated the intervention-mediator and mediator-outcome associations using linear or logistic regressions, as appropriate (Figure 1B). Examining these associations provides information on the magnitude and direction of the relationships between the intervention, mediator, and outcome, which is necessary for a meaningful interpretation of mediated effects estimated from the causal mediation analyses described below. All analyses were performed using a complete-case approach, given the proportion of missing data for each variable was low (≤5%).

All intervention-outcome, intervention-mediator, and mediator-outcome regression models were adjusted for the following covariates: traditional risk phenotype (low, high), age at baseline, sex (male, female), state of residence (dichotomized based on latitude: SA/VIC/TAS, QLD/NT/WA/NSW/ACT), socioeconomic status (Socio-Economic Indexes for Areas—Index of Relative Socio-Economic Advantage and Disadvantage in ­quintiles), education (school only, higher education), health literacy (low, high), risk taking behaviors (risk-seeking, risk-neutral, or risk-averse from the “Health and Safety” and “Social” domains from the Domain-Specific Risk-taking scale, DOSPERT),^13^^,^^14^ and baseline measures of mediator and/or outcome.^9^ Regression models assessing mediator-outcome associations additionally included the intervention group as a covariate. Inclusion of these pre-specified covariates ensured appropriate adjustment for potential confounding (for mediator-outcome associations) and consistency with the mediation analysis models. A sensitivity analysis using a reduced set of covariates (age, sex, phenotype, state of residence, and baseline measures of mediator and/or outcome) was performed to examine the stability of the analytical model and the robustness of results.

For psychosocial measures that were associated (*P* < .05) with both the intervention and a behavioral outcome, and for which the direction of associations was consistent with the overall intervention-outcome association, we quantified the extent to which the proposed mediator might explain the effect of the intervention on the outcome. Using the PARAMED module in Stata Statistical Software (Release 19),^15^ the total effect of the intervention on an outcome was decomposed into natural indirect effect (NIE) and natural direct effect (NDE). Under the counterfactual framework,^16^ the NIE represents the mediated effect for an individual and is defined as the difference in the outcome when the individual is set to receive the intervention and their mediator value is set to what it would have been under the intervention, compared with when the individual is set to receive the intervention and their mediator value is set to what it would have been without the intervention. The NDE represents the effect not operating through the mediator and is defined as the difference in outcome when the individual is set to receive the intervention compared with not receiving the intervention, while holding their mediator value at the level it would have been in the absence of the intervention. Bias-corrected bootstraps with 1000 resamples were used to estimate the 95% CIs for the NIE and NDE.

## Results

Baseline characteristics of study participants by study arms are presented for all participants, participants with high traditional melanoma risk, and women separately in Table 1. The intervention-mediator and mediator-outcome associations for each hypothesized psychosocial mediator and behavioral outcome are presented in Table 2, with associations that were statistically significant (*P* < .05) highlighted in bold text. These associations were used to identify suitable mediators for the subsequent mediation analysis. The effects mediated (NIEs) were formally quantified for psychosocial factors that were associated with both the intervention and a behavioral outcome, and for which the direction of associations was consistent with the overall intervention-outcome association. The estimated NIE and NDE for potential mediators are presented in Table 3. Results from the sensitivity analysis using a reduced set of covariates were consistent with results from the primary analysis and are presented in Tables S2 and S3.

**Table 1:** Baseline characteristics of participants by study arm and population subgroup.

	All participants (*N =* 1024)		High traditional risk (*N =* 505)		Women (*N =* 521)	
	Intervention (*N =* 513)	Control (*N =* 511)	Intervention (*N =* 252)	Control (*N =* 253)	Intervention (*N =* 261)	Control (*N =* 260)
**Age, mean (SD)**	47.1 (14.8)	46.7 (14.3)	48.2 (14.0)	47.9 (13.9)	46.7 (14.0)	45.5 (14.5)
**Sex, *N* (%)**						
** Male**	252 (49.1%)	251 (49.1%)	121 (48.0%)	119 (47.0%)	0 (0.0%)	0 (0.0%)
** Female**	261 (50.9%)	260 (50.9%)	131 (52.0%)	134 (53.0%)	261 (100.0%)	260 (100.0%)
**Phenotype, *N* (%)**						
** High traditional risk**	261 (50.9%)	258 (50.5%)	252 (100.0%)	253 (100.0%)	130 (49.8%)	126 (48.5%)
** Low traditional risk**	252 (49.1%)	253 (49.5%)	0 (0.0%)	0 (0.0%)	131 (50.2%)	134 (51.5%)
**State of residence, *N* (%)**						
** SA/VIC/TAS**	197 (38.4%)	197 (38.6%)	82 (32.5%)	80 (31.6%)	101 (38.7%)	111 (42.7%)
** QLD/NT/WA/NSW/ACT**	316 (61.6%)	314 (61.4%)	170 (67.5%)	173 (68.4%)	160 (61.3%)	149 (57.3%)
**Socioeconomic status, *N* (%)**						
** 1 (most disadvantaged)**	98 (19.3%)	107 (21.2%)	41 (16.4%)	50 (20.1%)	47 (18.1%)	53 (20.7%)
** 2**	115 (22.6%)	89 (17.7%)	59 (23.6%)	44 (17.7%)	65 (25.1%)	42 (16.4%)
** 3**	97 (19.1%)	102 (20.2%)	54 (21.6%)	56 (22.5%)	48 (18.5%)	56 (21.9%)
** 4**	109 (21.4%)	95 (18.8%)	49 (19.6%)	47 (18.9%)	54 (20.8%)	51 (19.9%)
** 5**	90 (17.7%)	111 (22.0%)	47 (18.8%)	52 (20.9%)	45 (17.4%)	54 (21.1%)
**Education, *N* (%)**						
** School**	120 (23.4%)	119 (23.3%)	52 (20.6%)	58 (22.9%)	47 (18.0%)	57 (21.9%)
** Higher education**	393 (76.6%)	392 (76.7%)	200 (79.4%)	195 (77.1%)	214 (82.0%)	203 (78.1%)
**Health literacy, *N* (%)**						
** Low**	66 (12.9%)	49 (9.6%)	30 (11.9%)	21 (8.3%)	20 (7.7%)	17 (6.5%)
** High**	447 (87.1%)	462 (90.4%)	222 (88.1%)	232 (91.7%)	241 (92.3%)	243 (93.5%)
**Health risk-taking, *N* (%)**						
** Risk-averse**	64 (12.5%)	71 (13.9%)	28 (11.1%)	35 (13.8%)	48 (18.4%)	48 (18.5%)
** Risk-neutral**	374 (72.9%)	351 (68.7%)	193 (76.6%)	181 (71.5%)	189 (72.4%)	187 (71.9%)
** Risk-seeking**	75 (14.6%)	89 (17.4%)	31 (12.3%)	37 (14.6%)	24 (9.2%)	25 (9.6%)
**Social risk-taking, *N* (%)**						
** Risk-averse**	96 (18.7%)	91 (17.8%)	33 (13.1%)	54 (21.3%)	57 (21.8%)	43 (16.5%)
** Risk-neutral**	315 (61.4%)	327 (64.0%)	169 (67.1%)	156 (61.7%)	153 (58.6%)	164 (63.1%)
** Risk-seeking**	102 (19.9%)	93 (18.2%)	50 (19.8%)	43 (17.0%)	51 (19.5%)	53 (20.4%)

**Table 2:** Intervention-mediator and mediator-outcome associations for hypothesized psychosocial mediators by outcome of interest.

	Intervention-mediator^a^	Mediator-outcome^a^
**SPH at T1 among the high traditional risk group (*N* = 482)**	Mean difference (95% CI)	Mean difference (95% CI)
** Perceived severity of melanoma**	−0.02 (−0.13, 0.09)	0.02 (−0.04, 0.09)
** Perceived absolute risk of melanoma**	−**13.85 (**−**17.47 to** −**10.23)**	−**0.00 (**−**0.00 to** −**0.00)**
** Perceived relative risk of melanoma**	−0.10 (−0.23 to 0.02)	−0.01 (−0.06 to 0.04)
** Perceived control over melanoma development**	−0.04 (−0.19 to 0.11)	0.04 (−0.01 to 0.08)
** Perceived effectiveness of SPH**	**0.13 (0.03 to 0.23)**	**0.10 (0.03 to 0.16)**
** Perceived importance of SPH**	**0.09 (0.02 to 0.17)**	**0.20 (0.11 to 0.29)**
** Perceived capability for SPH**	0.02 (−0.06 to 0.10)	**0.15 (0.07 to 0.23)**
** Family communication on SPH**	0.06 (−0.10 to 0.22)	**0.16 (0.12 to 0.20)**
** Tanning attitudes (Pro-tan score)**	−0.22 (−0.83 to 0.40)	−**0.01 (**−**0.02 to** −**0.00)**
** Worry about Vitamin D deficiency**	−0.02 (−0.10 to 0.05)	−0.06 (−0.15 to 0.03)
**Intentional tanning at T1 among women (*N* = 499)**	Mean difference (95% CI)	Mean difference (95% CI)
** Perceived severity of melanoma**	−0.03 (−0.14 to 0.08)	**0.08 (0.00 to 0.16)**
** Perceived absolute risk of melanoma**	−**14.46 (**−**17.88 to** −**11.04)**	0.00 (−0.00 to 0.00)
** Perceived relative risk of melanoma**	−**0.17 (**−**0.29 to** −**0.04)**	0.00 (−0.07 to 0.07)
** Perceived control over melanoma development**	0.09 (−0.04 to 0.23)	0.01 (−0.06 to 0.07)
** Perceived effectiveness of SPH**	**0.13 (0.03 to 0.22)**	−0.03 (−0.12 to 0.06)
** Perceived importance of SPH**	0.06 (−0.00 to 0.13)	−**0.15 (**−**0.27 to** −**0.02)**
** Perceived capability for SPH**	0.01 (−0.07 to 0.09)	−0.09 (−0.20 to 0.02)
** Family communication on SPH**	0.12 (−0.06 to 0.29)	0.04 (−0.01 to 0.09)
** Tanning attitudes (Pro-tan score)**	−0.42 (−1.09 to 0.25)	**0.03 (0.01 to 0.04)**
** Worry about Vitamin D deficiency**	−0.06 (−0.14 to 0.01)	0.03 (−0.08 to 0.15)
**WSE at T1 among women (*N* = 499)**	Mean difference (95% CI)	Odds ratios (95% CI)
** Perceived severity of melanoma**	−0.03 (−0.14 to 0.08)	0.94 (0.66 to 1.34)
** Perceived absolute risk of melanoma**	−**14.46 (**−**17.88 to** −**11.04)**	1.00 (0.99 to 1.01)
** Perceived relative risk of melanoma**	−**0.17 (**−**0.29 to** −**0.04)**	0.90 (0.66 to 1.23)
** Perceived control over melanoma development**	0.09 (−0.04 to 0.23)	1.11 (0.82 to 1.52)
** Perceived importance of WSE**	0.06 (−0.02 to 0.15)	1.60 (0.95 to 2.71)
** Perceived capability for WSE**	**0.10 (0.01 to 0.19)**	**2.33 (1.39 to 3.92)**
** Family communication on WSE**	0.03 (−0.14 to 0.21)	**1.87 (1.48 to 2.37)**
** Perceived control over melanoma detection**	**0.21 (0.07 to 0.35)**	**1.91 (1.39 to 2.63)**
** Confidence identifying melanoma**	0.08 (−0.07 to 0.22)	**2.24 (1.65 to 3.05)**
** SSEAS score**	0.35 (−0.31 to 1.01)	**1.23 (1.13 to 1.33)**
** Anxiety about WSE**	−0.14 (−0.28 to 0.01)	0.91 (0.68 to 1.21)
**Sunburn at T2 (*N* = 961)**	Mean difference (95% CI)	Odds ratios (95% CI)
** Perceived severity of melanoma**	0.01 (−0.07 to 0.08)	1.00 (0.70 to 1.42)
** Perceived absolute risk of melanoma**	−**13.89 (**−**16.37 to** −**11.41)**	**1.01 (1.00 to 1.02)**
** Perceived relative risk of melanoma**	−0.08 (−0.17 to 0.02)	1.28 (0.96 to 1.69)
** Perceived control over melanoma development**	0.01 (−0.09 to 0.11)	0.84 (0.65 to 1.09)
** Perceived effectiveness of SPH**	0.06 (−0.01 to 0.13)	0.83 (0.58 to 1.18)
** Perceived importance of SPH**	0.03 (−0.02 to 0.08)	0.89 (0.54 to 1.45)
** Perceived capability for SPH**	−0.00 (−0.06 to 0.05)	**0.63 (0.40 to 0.99)**
** Family communication on SPH**	0.08 (−0.04 to 0.20)	1.01 (0.81 to 1.26)
** Tanning attitudes (Pro-tan score)**	−0.09 (−0.53 to 0.35)	1.05 (0.99 to 1.11)
** Worry about Vitamin D deficiency**	−0.03 (−0.08 to 0.02)	1.04 (0.63 to 1.72)

Abbreviations: CI = confidence interval; SPH = sun protection habits; SSEAS = Skin Self-Examination Attitude Scale; WSE = whole-body skin examination.

a Estimates in bold font indicates *P* < .05.

**Table 3:** Estimated natural indirect effects for potential mediators of intervention’s effect on melanoma prevention or early detection behavioral outcomes.

Outcome	Mediator				
**SPH at T1 among the high traditional risk group (*N* = 482)**		TE^a^	0.13 (0.06-0.20)		
	Perceived importance of SPH	NIE^a^	0.02 (0.00-0.04)^b^	NDE^a^	0.11 (0.03-0.18)^b^
	Perceived effectiveness of SPH	NIE^a^	0.01 (0.00-0.03)^b^	NDE^a^	0.12 (0.05-0.19)^b^
**WSE at T1 among women (*N* = 499)**		TE^c^	1.83 (1.15-2.91)		
	Perceived capability for WSE	NIE^c^	1.08 (1.00-1.29)^b^	NDE^c^	1.67 (0.94-2.64)^b^
	Perceived control over melanoma detection	NIE^c^	1.13 (1.03-1.32)^b^	NDE^c^	1.55 (0.95-2.52)^b^
**Sunburn at T2 (*N* = 961)**		TE^c^	0.62 (0.41-0.94)		
	Perceived absolute risk of melanoma	NIE^c^	0.92 (0.75-1.11)^b^	NDE^c^	0.66 (0.42-1.05)

Abbreviations: NDE = natural direct effect; NIE = natural indirect effect; SPH = sun protection habits; TE = total effect; WSE = whole-body skin examination.

a Estimated mean difference and 95% confidence interval.

b Bias-corrected bootstrapped 95% confidence intervals with 1000 replications.

c Estimated odds ratios and 95% confidence interval.

### Sun protection habits at T1 among participants with high traditional melanoma risk

Among participants with high traditional melanoma risk, receiving personal melanoma genomic risk information improved sun protection habits at T1 (mean difference in total index score 0.13; 95% CI: 0.06-0.20). The intervention increased perceived effectiveness (mean difference in average combined score 0.13; 95% CI: 0.03-0.23) and perceived importance (0.09; 95% CI: 0.02-0.17) of sun protection habits in reducing melanoma risk at T1. Perceived effectiveness (mean difference in average combined score 0.10; 95% CI: 0.03-0.16) and perceived importance (0.20; 95% CI: 0.11-0.29) were positively associated with sun protection habits at T1. In mediation analysis, perceived importance (NIE mean difference 0.02; 95% CI: 0.00-0.04) and perceived effectiveness (0.01; 0.00-0.03) of sun protection habits partially mediated the intervention’s effect on increased sun protection behaviors.

### Intentional tanning at T1 among women

Among women, receiving personal melanoma genomic risk information reduced intentional tanning at T1 (mean difference −0.12; 95% CI: −0.22 to −0.02). No psychosocial factor was associated with both the intervention and intentional tanning frequency, and thus no further mediation analysis was performed.

### Whole-body skin examination at T1 among women

Among women, receiving personal melanoma genomic risk information increased whole-body skin examinations at T1 (odds ratio [OR] 1.83; 95% CI: 1.15-2.91). The intervention increased perceived capability to engage in whole-body skin examinations (mean difference 0.13; 95% CI: 0.02-0.24) and perceived control over detecting a future melanoma early in its development (0.21; 95% CI: 0.07-0.35) at T1. Perceived capability to engage in whole-body skin examinations (OR 2.33; 95% CI: 1.39-3.92) and perceived control over detecting a future melanoma (1.91; 1.39-2.63) were positively associated with whole-body skin examinations at T1. In mediation analysis, perceived capability to engage in skin examinations (NIE OR 1.08; 95% CI: 1.00-1.29) and perceived control over detecting a future melanoma (1.13; 1.03-1.32) partially mediated the intervention’s effect on increased whole-body skin examinations.

### Sunburn at T2

Receiving personal melanoma genomic risk information reduced self-reported sunburn incidence at T2 (OR 0.62; 95% CI: 0.41-0.94), most evidently among participants in the high traditional risk group (OR 0.41; 95% CI: 0.22-0.76) and among women (OR 0.36; 95% CI: 0.18-0.70). The intervention reduced perceived absolute risk of melanoma (mean difference on a 0-100 scale −13.89; 95% CI: −16.37 to −11.41), which was positively associated with odds of sunburn (OR 1.01; 95% CI: 1.00-1.02). In mediation analysis, there was no evidence that perceived absolute risk of melanoma mediated the intervention’s effect on reduced sunburn (NIE OR 0.92; 95% CI: 0.75-1.11). Consistent results were observed among participants in the high traditional risk group (perceived absolute risk of melanoma NIE OR 1.05; 95% CI: 0.74-1.55) and among women (0.94; 0.76-1.02).

### Exploratory analyses

In the exploratory analysis evaluating individual sun protection habits and types of skin examination separately, we identified several potential mediators: (1) the intervention increased perceived effectiveness of protective clothing (mean difference 0.17; 95% CI: 0.04-0.30), which was positively associated with sun protection habits (0.08; 0.03-0.13) at T1 among participants in the high traditional risk group, the NIE was 0.01 (95% CI: 0.00-0.03) indicating partial mediation; (2) the intervention increased perceived importance of wearing long pants (mean difference 0.13; 95% CI: 0.01-0.26), which was positively associated with sun protection habits (0.09; 0.04-0.15) at T1 among participants in the high traditional risk group, the NIE was 0.01 (95% CI: 0.00-0.04) indicating partial mediation; (3) the intervention increased perceived effectiveness of limiting midday sun exposure (mean difference 0.11; 95% CI: 0.00-0.23), which was inversely associated with intentional tanning (−0.08; −0.16 to −0.00) at T1 among women, the NIE was 0.00 (95% CI: −0.02 to 0.01) indicating no mediation; (4) the intervention increased perceived capability to engage in skin self-examinations (mean difference 0.13; 95% CI: 0.02-0.24), which was positively associated with whole-body skin examinations (OR 1.97; 95% CI: 1.31-2.95) at T1 among women, the NIE OR was 1.12 (95% CI: 1.01-3.17) indicating partial mediation; (5) the intervention increased perceived importance of staying in the shade (mean difference 0.10; 95% CI: 0.00-0.19), which was inversely associated with sunburn (OR 0.52; 95% CI: 0.29-0.95) at T2 among women, the NIE OR was 0.94 (95% CI: 0.76-1.02) indicating no mediation.

## Discussion

We evaluated the hypothesized psychosocial mediators for 4 behavioral outcomes observed in the Melanoma Genomics Managing Your Risk Study. Our key findings were: (1) increased perceived importance and increased perceived effectiveness of sun protection habits may have partly mediated the effect of receiving personal melanoma risk information on increased sun protection behaviors among participants with high traditional melanoma risk; (2) increased perceived capability to engage in skin examinations and increased perceived control over detecting a future melanoma may have partly mediated the intervention’s effect on increased whole-body skin examinations among women; (3) we did not find any psychosocial mediators for the intervention’s effect on reduced intentional tanning among women; (4) perceived absolute risk of melanoma was identified as a potential mediator for the intervention’s effect on reduced self-reported sunburn for all study participants, but results from the mediation analysis suggested it did not mediate the observed effect.

The estimated NIEs were modest in magnitude, suggesting that the psychosocial factors measured in this study may explain only a small proportion of the intervention’s effects on melanoma prevention and early detection behaviors. This finding is not unexpected given that health behavior change is typically influenced by multiple determinants, and mediation requires both an association between the psychosocial factor and the behavioral outcome and an effect of the intervention on that psychosocial factor. As shown in Table 2, many of the hypothesized psychosocial factors were associated with the relevant behaviors; however, the intervention had relatively limited effects on several of these constructs. These findings suggest that the behavioral effects of personal genomic risk information may operate through multiple pathways, only some of which were captured by the psychosocial constructs examined in this analysis.

We also found no evidence that the measured psychosocial factors mediated the intervention’s effects on reduced intentional tanning among women or reduced sunburn. These findings suggest that the Protection Motivation Theory and Health Belief Model examined in this study may explain some, but not all, pathways through which genomic risk information influences behavior. In particular, intentional tanning may be driven by determinants that are less well captured by these frameworks, including appearance-related motivations, emotional factors, and sociocultural influences.^17^ Cognitive Dissonance Theory has also been proposed to explain how individuals may rationalize tanning behaviors despite awareness of associated risks.^17^ Likewise, sunburn risk may depend not only on engagement in sun protection behaviors but also on their consistency, timing, and correct implementation, factors that may not be adequately reflected in the psychosocial determinants measured.^18^^,^^19^ Taken together, these null mediation findings highlight the possibility that additional theoretical perspectives and contextual determinants are needed to fully understand the mechanisms through which genomic risk information influences prevention and early detection behaviors. Future studies should therefore investigate a broader range of behavioral theories and contextual factors to elucidate these pathways.

The mediation analysis assumes there are no unmeasured confounding of intervention-outcome, intervention-mediator, and mediator-outcome relationships, and no intervention-induced mediator-outcome confounding.^16^ Given the intervention was randomized, the assumptions of unconfounded intervention-outcome and intervention-mediator relationships were satisfied. For mediator-outcome associations, our analysis accounted for a comprehensive set of baseline demographic, social, and behavioral factors, although residual confounding from unmeasured factors cannot be ruled out.

To ensure the intervention preceded psychosocial and behavioral changes, we used prospective data for the analyses of intervention-mediator and intervention-outcome associations. For the analysis of association between psychosocial measures and behavioral outcomes observed at T1, cross-sectional data were used. While we assumed that changes in psychosocial factors preceded changes in behavior, the temporal ordering necessary to support causal inference could not be established. Accordingly, these findings should be interpreted with caution. For the analysis of association between psychosocial measures and sunburn observed at T2, prospective data were used.

It is difficult to disentangle the independent effects of receiving personalized genomic risk information and genetic counseling on psychosocial and behavioral changes. Alongside delivery of a personalized booklet with their risk information and how they could manage their risk, participants in the intervention arm received one-on-one telephone-based genetic counseling (average duration 7 mins, range 2-25 mins), during which they were offered the opportunity to ask questions regarding sun protection and skin examination after receiving their genomic risk results. The most prevalent discussion topics during the counseling call related to past sun exposure and past and current sun habits,^20^ and these conversations may also have affected participants’ psychosocial measures. Qualitative interviews with a subgroup of participants from the main trial indicated that the effect contributed by telephone counseling may have varied by genomic risk level. Some participants who received average or low genomic risk results perceived the telephone counseling as providing limited additional benefit beyond the personalized booklet.^20^

As observed in the main trial, the impact of providing personal genomic risk information on health-related behavioral outcomes varied across population subgroups.^5^ Although reductions in sunburn were observed among all participants, other prevention and early detection behaviors were evident only within specific subgroups, thereby limiting the generalizability of the findings to the whole population. Notably, these subgroups, namely participants with high traditional risk and women, who were more likely than men to engage in intentional tanning,^5^ represent populations at higher risk for melanoma, who may benefit the most from receiving personal genomic risk information. Personal risk communication strategies targeting these populations may thus be further optimized by addressing the psychosocial mediators identified in this study.

Our findings indicate that perceived importance and perceived effectiveness of sun protection habits, most evidently for wearing protective clothing, may have partially mediated the effect of receiving personal melanoma risk information on increased sun protection behaviors among participants with high traditional melanoma risk. These findings are in line with prior research among individuals with a strong family history of melanoma, where genetic testing for *CDKN2A/p16* was associated with sustained improvements in sun protection behaviors from 1 month up to 2 years post-testing, with the greatest improvements observed for wearing photoprotective clothing.^21^^,^^22^ Similarly, a previous randomized trial on providing personal melanoma genomic risk information to individuals with a strong family history reported a statistically significant difference between the intervention and control arm in wearing long-sleeved shirts.^23^ Collectively, the existing body of evidence suggests that compared to other sun protection behaviors, such as regular sunscreen reapplication or consistent shade seeking, wearing protective clothing may represent a comparatively easier sun protection behavior to adopt.

Receiving personal melanoma risk information increased perceived capability to engage in skin examinations and perceived control over detecting a future melanoma, which may have partially mediated the intervention’s effect on increased whole-body skin examinations among women. These findings are consistent with existing evidence suggesting that increased knowledge and confidence may facilitate skin examination behaviors. In a qualitative study, 19 of 30 participants reported satisfaction with genomic testing, with gaining new knowledge about their melanoma risk or about preventive behaviors identified as a key reason.^8^ Participants across all genomic risk groups who perceived knowledge gain also reported feeling better equipped to manage their skin cancer risk.^8^ Similarly, a U.S. population-based cross-sectional survey found that higher levels of melanoma knowledge and confidence in performing skin self-examination were positively associated with skin self-examination behavior.^24^

Receiving personal genomic melanoma risk information led to reduced perceived absolute risk of melanoma, in line with existing evidence that most individuals overestimate their risk of cancer.^25^ Reduced perceived absolute risk of melanoma was also identified as a potential mediator of the intervention’s effect on reduced self-reported sunburn, but results from the mediation analysis suggest perceived risk of melanoma did not mediate the observed effect. Similarly, there was no evidence that the intervention’s effect on reduced intentional tanning among women was mediated by any of the psychosocial mediators evaluated, suggesting the effect of the intervention on observed outcomes was mediated by unmeasured pathways. Given sunburn and intentional tanning are key risk factors for melanoma, future studies aimed to evaluate other possible mediating pathways for intervention’s effect on reduced intentional tanning and sunburn are warranted.

## Supplementary Material

kaag046_Supplementary_Data

## Transparency statement

The Study Protocol (DOI: 10.1016/j.cct.2018.05.014) and Statistical Analysis Plan (DOI: 10.1186/s13063-020-04351-w) for the parent trial have been published. De-identified data from this study are not available in a public archive. Requests for obtaining de-identified data should be addressed to the corresponding author. Analytic code used to conduct the analyses presented in this study are not available in a public archive. They may be made available by emailing the corresponding author. Materials used to conduct the study are not publicly available.
