# Supplementary material for: Psychosocial mediators for the impact of personal genomic risk information on melanoma prevention and early detection behaviors
Source: Ann Behav Med. 2026 Aug 26;60(1):kaag046. doi: 10.1093/abm/kaag046 (PMC13509948; doi:10.1093/abm/kaag046)
Supplement: Supplementary file 1 [file kaag046_supplementary_data.docx]

Table S1	Estimates of intervention-outcome effects derived from main trial and from the present mediation analysis

|  | **Intervention-outcome effect estimates** | |
| --- | --- | --- |
| **Outcome** | **Primary trial** | **Current analysis** |
| **SPH at T1 among the high traditional risk group (N=482)** | Mean difference (95% CI)^1^  0.12 (0.05, 0.19) | Mean difference (95% CI)^3^  0.13 (0.06, 0.20) |
| **Intentional tanning at T1 among women (N=499)** | Mean difference (95% CI)^1^  −0.12 (−0.22, −0.03) | Mean difference (95% CI)^3^  -0.12 (-0.22, -0.02) |
| **WSE at T1 among women (N=499)** | Risk ratio (95% CI)^2^  1.57 (1.11, 2.22) | Odds ratio (95% CI)^4^  1.83 (1.15, 2.91) |
| **Sunburn at T2 (N=961)** | Risk ratio (95% CI)^2^  0.72 (0.54, 0.96) | Odds ratio (95% CI)^4^  0.62 (0.41, 0.94) |

^1^ Generalized linear mix models (GLMMs) adjusted for all randomised stratification factors (i.e., State, age group, and sex), baseline covariate & includes treatment by FUP interaction

^2^ Generalized estimating equations (GEE) with a log link function adjusted for all randomised stratification factors (i.e., State (dichotomized based on latitude: SA/VIC/TAS, QLD/NT/WA/NSW/ACT), age group, and sex), baseline covariate and treatment by FUP interaction

^3^ Linear regression adjusted for all randomised stratification factors (i.e., State (dichotomized based on latitude: SA/VIC/TAS, QLD/NT/WA/NSW/ACT), age group, and sex), baseline covariate, socioeconomic status (Socio-Economic Indexes for Areas - Index of Relative Socio-Economic Advantage and Disadvantage in quintiles), education (school only, higher education), health literacy (low, high), risk taking behaviors [risk-seeking, risk-neutral, or risk-averse from the ‘Health and Safety’ and ‘Social’ domains from the Domain-Specific Risk-taking scale (DOSPERT)]

^4^ Logistic regression adjusted for all randomised stratification factors (i.e., State (dichotomized based on latitude: SA/VIC/TAS, QLD/NT/WA/NSW/ACT), age group, and sex), baseline covariate, socioeconomic status (Socio-Economic Indexes for Areas - Index of Relative Socio-Economic Advantage and Disadvantage in quintiles), education (school only, higher education), health literacy (low, high), risk taking behaviors [risk-seeking, risk-neutral, or risk-averse from the ‘Health and Safety’ and ‘Social’ domains from the Domain-Specific Risk-taking scale (DOSPERT)]

CI = confidence intervals SPH = sun protection habits; WSE = whole-body skin examination

Table S2	Intervention-mediator and mediator-outcome associations for hypothesized psychosocial mediators by outcome of interest - estimates from statistical model including a minimal set of covariates (age, sex, phenotype, state of residence, baseline score of the mediator [and outcome for mediator-outcome associations])

|  | **Intervention-mediator^1^** | **Mediator-outcome^1^** |
| --- | --- | --- |
| ***SPH at T1 among the high traditional risk group (N=488)*** | *Mean difference (95% CI)* | *Mean difference (95% CI)* |
| Perceived severity of melanoma | -0.00 (-0.11, 0.11) | 0.03 (-0.03, 0.10) |
| Perceived absolute risk of melanoma | **-14.19 (-17.73, -10.65)** | **-0.00 (-0.00, -0.00)** |
| Perceived relative risk of melanoma | -0.12 (-0.24, 0.00) | -0.01 (-0.06, 0.04) |
| Perceived control over melanoma development | -0.01 (-0.16, 0.13) | 0.04 (-0.00, 0.09) |
| Perceived effectiveness of SPH | 0.08 (-0.09, 0.24) | **0.17 (0.13, 0.20)** |
| Perceived importance of SPH | **0.14 (0.04, 0.24)** | **0.10 (0.03, 0.16)** |
| Perceived capability for SPH | **0.10 (0.03, 0.18)** | **0.22 (0.13, 0.30)** |
| Family communication on SPH | 0.02 (-0.06, 0.10) | **0.17 (0.08, 0.25)** |
| Tanning attitudes (Pro-tan score) | -0.26 (-0.87, 0.35) | -0.01 (-0.02, 0.00) |
| Worry about Vitamin D deficiency | -0.02 (-0.09, 0.06) | -0.08 (-0.16, 0.01) |
| ***Intentional tanning at T1 among women (N=505)*** | *Mean difference (95% CI)* | *Mean difference (95% CI)* |
| Perceived severity of melanoma | -0.02 (-0.13, 0.09) | 0.08 (-0.00, 0.15) |
| Perceived absolute risk of melanoma | **-14.82 (-18.18, -11.46)** | 0.00 (-0.00, 0.00) |
| Perceived relative risk of melanoma | **-0.15 (-0.28, -0.03)** | -0.00 (-0.07, 0.07) |
| Perceived control over melanoma development | 0.09 (-0.05, 0.22) | 0.01 (-0.05, 0.08) |
| Perceived effectiveness of SPH | 0.11 (-0.06, 0.28) | 0.04 (-0.01, 0.09) |
| Perceived importance of SPH | **0.11 (0.01, 0.20)** | -0.04 (-0.13, 0.05) |
| Perceived capability for SPH | 0.06 (-0.01, 0.13) | **-0.16 (-0.28, -0.03)** |
| Family communication on SPH | 0.00 (-0.08, 0.08) | -0.10 (-0.21, 0.01) |
| Tanning attitudes (Pro-tan score) | -0.33 (-0.99, 0.32) | **0.03 (0.01, 0.04)** |
| Worry about Vitamin D deficiency | -0.05 (-0.13, 0.02) | 0.01 (-0.10, 0.13) |
| ***WSE at T1 among women (N=505)*** | *Mean difference (95% CI)* | *Odds ratios (95% CI)* |
| Perceived severity of melanoma | -0.02 (-0.13, 0.09) | 0.94 (0.66, 1.33) |
| Perceived absolute risk of melanoma | **-14.82 (-18.18, -11.46)** | 1.00 (0.99, 1.01) |
| Perceived relative risk of melanoma | **-0.15 (-0.28, -0.03)** | 0.91 (0.67, 1.24) |
| Perceived control over melanoma development | 0.09 (-0.05, 0.22) | 1.11 (0.82, 1.50) |
| Perceived importance of WSE | 0.06 (-0.03, 0.14) | 1.56 (0.94, 2.60) |
| Perceived capability for WSE | **0.10 (0.01, 0.19)** | **2.23 (1.35, 3.70)** |
| Family communication on WSE | 0.06 (-0.11, 0.23) | **1.88 (1.49, 2.37)** |
| Perceived control over melanoma detection | **0.21 (0.07, 0.35)** | **1.83 (1.35, 2.48)** |
| Confidence identifying melanoma | 0.07 (-0.07, 0.21) | **2.09 (1.56, 2.81)** |
| SSEAS score | 0.33 (-0.31, 0.97) | **1.22 (1.13, 1.32)** |
| Anxiety about WSE | -0.12 (-0.26, 0.02) | 0.92 (0.69, 1.21) |
| ***Sunburn at T2 (N=994)*** | *Mean difference (95% CI)* | *Odds ratios (95% CI)* |
| Perceived severity of melanoma | 0.01 (-0.07, 0.09) | 0.94 (0.67, 1.32) |
| Perceived absolute risk of melanoma | **-13.99 (-16.46, -11.53)** | **1.01 (1.00, 1.02)** |
| Perceived relative risk of melanoma | -0.08 (-0.18, 0.01) | 1.31 (1.00, 1.73) |
| Perceived control over melanoma development | 0.01 (-0.09, 0.10) | 0.85 (0.66, 1.09) |
| Perceived effectiveness of SPH | 0.08 (-0.03, 0.20) | 1.00 (0.81, 1.24) |
| Perceived importance of SPH | 0.08 (-0.01, 0.17) | 0.78 (0.61, 1.00) |
| Perceived capability for SPH | 0.03 (-0.02, 0.08) | 0.79 (0.49, 1.27) |
| Family communication on SPH | -0.01 (-0.06, 0.05) | **0.58 (0.37, 0.90)** |
| Tanning attitudes (Pro-tan score) | -0.08 (-0.52, 0.35) | 1.05 (0.99, 1.11) |
| Worry about Vitamin D deficiency | -0.02 (-0.07, 0.03) | 1.02 (0.62, 1.66) |

^1^ Estimates in bold font indicates p<0.05

CI = confidence interval; SPH = sun protection habits; SSEAS = Skin Self-Examination Attitude Scale; WSE = whole-body skin examination

Table S3	Estimated natural indirect effects for potential mediators of intervention’s effect on melanoma prevention or early detection behavioral outcomes - estimates from statistical model including a minimal set of covariates (age, sex, phenotype, state of residence, baseline score of the mediator [and outcome for mediator-outcome associations])

| ***Outcome*** | Mediator |  |  |  |  |
| --- | --- | --- | --- | --- | --- |
| ***SPH at T1 among the high traditional risk group (N=482)*** |  | ***TE^1^*** | ***0.13 (0.05, 0.20)*** |  |  |
|  | Perceived importance of SPH | NIE^1^ | 0.02 (0.01, 0.05)^3^ | NDE^1^ | 0.11 (0.03, 0.18)^3^ |
|  | Perceived effectiveness of SPH | NIE^1^ | 0.01 (0.00, 0.03)^3^ | NDE^1^ | 0.12 (0.05, 0.19)^3^ |
| ***WSE at T1 among women (N=499)*** |  | ***TE^2^*** | ***1.91 (1.22, 3.02)*** |  |  |
|  | Perceived capability for WSE | NIE^2^ | 1.07 (1.00, 1.26)^3^ | NDE^2^ | 1.76 (1.09, 2.75)^3^ |
|  | Perceived control over melanoma detection | NIE^2^ | 1.12 (1.04, 1.29)^3^ | NDE^2^ | 1.66 (0.99, 2.53)^3^ |
| ***Sunburn at T2 (N=961)*** |  | ***TE^2^*** | ***0.66 (0.44, 0.99)*** |  |  |
|  | Perceived absolute risk of melanoma | NIE^2^ | 0.90 (0.74, 1.09)^3^ | NDE^2^ | 0.72 (0.45, 1.10)^3^ |

^1^ Estimated mean difference and 95% confidence interval

^2^ Estimated odds ratios and 95% confidence interval

^3^ Bias-corrected bootstrapped 95% confidence intervals with 1000 replications

NDE = natural direct effect; NIE = natural indirect effect; SPH = sun protection habits; TE = total effect; WSE = whole-body skin examination
